# Zukunft der akutstationären Rheumatologie in Deutschland: Stellungnahme der DGRh- und VRA-Vorstände zur Krankenhausplanung NRW 2019 für den Leistungsbereich Rheumatologie

**DOI:** 10.1007/s00393-020-00939-4

**Published:** 2020-12-12

**Authors:** H.-J. Lakomek, A. Krause, J. Braun, B. Hellmich, M. Klass, H. Lorenz, M. Schneider, H. Schulze-Koops, C. Specker

**Affiliations:** 1 Universitätsklinik für Geriatrie, Mühlenkreiskliniken, Johannes Wesling Klinikum Minden, Hans-Nolte-Str. 1, 32429 Minden, Deutschland; 2 Abteilung Rheumatologie, Klinische Immunologie und Osteologie, Immanuel Krankenhaus Berlin, Berlin, Deutschland; 3 Rheumazentrum Ruhrgebiet und Ruhr-Universität Bochum, Herne, Deutschland; 4 Vaskulitiszentrum Süd, Klinik für Innere Medizin, Rheumatologie und Immunologie, Medius Kliniken – Akademisches Lehrkrankenhaus der Universität Tübingen, Kirchheim u. Teck, Deutschland; 5 Klinik für Rheumatologie und Physikalische Therapie, Helios St. Johannes Klinik, Duisburg, Deutschland; 6 Sektion Rheumatologie, Med. Klinik V, Universitätsklinikum Heidelberg, Heidelberg, Deutschland; 7 ACURA-Rheumazentrum Baden-Baden, Baden-Baden, Deutschland; 8 Poliklinik, Funktionsbereich und Hiller-Forschungszentrum für Rheumatologie, Heinrich-Heine-Universität Düsseldorf, Düsseldorf, Deutschland; 9 Sektion Rheumatologie und Klinische Immunologie, Medizinische Klinik IV, Ludwig-Maximilians-Universität München, München, Deutschland; 10 Klinik für Rheumatologie und Klinische Immunologie, Evang. Krankenhaus Essen-Werden, Kliniken Essen-Mitte, Essen, Deutschland

**Keywords:** Bedarfsplanung, Leistungsbereiche, Leistungsgruppen, Ministerium für Arbeit, Gesundheit und Soziales, Ambulantisierungspotenzial, Requirements planning, Disciplines, Organizational groups, Ministry of Labor, Health and Welfare, Outpatient potential

## Abstract

Im September 2019 wurde vom Ministerium für Arbeit, Gesundheit und Soziales (MAGS) in NRW ein Gutachten zur Krankenhausplanung veröffentlicht. Hierin wurde eine grundlegende Reform der Krankenhausplanung empfohlen, indem zukünftig eine Bedarfsplanung auf der Grundlage einer detaillierten Ausweisung von Leistungsbereichen und Leistungsgruppen erfolgen soll. Nach Aufforderung durch das MAGS NRW hat auch die Deutsche Gesellschaft für Rheumatologie (DGRh) mit Unterstützung des Verbandes Rheumatologischer Akutkliniken (VRA) hierzu Stellung genommen.

**Zusatzmaterial online:**

Die Online-Version dieses Beitrags (10.1007/s00393-020-00939-4) enthält eine Tabelle zur Zuordnung von Qualitätsvorgaben für Leistungsgruppen. Beitrag und Zusatzmaterial stehen Ihnen auf www.springermedizin.de zur Verfügung. Bitte geben Sie dort den Beitragstitel in die Suche ein, das Zusatzmaterial finden Sie beim Beitrag unter „Ergänzende Inhalte“. 
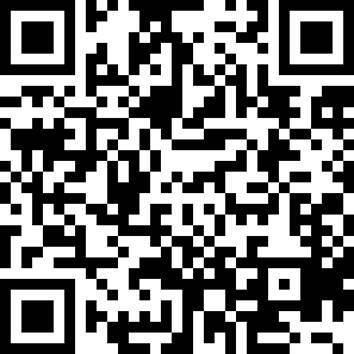

## Abstract (en)

In September 2019 the Ministry of Labor, Health and Welfare (MAGS) of North-Rhine/Westphalia (NRW) published an expert report on hospital planning. In this report a fundamental reform of hospital planning was recommended, in that a requirements planning should be carried out in the future on the basis of a detailed designation of disciplines and organizational groups. At the request of the MAGS NRW, the German Society for Rheumatology (DGRh) with the support of the Association of Rheumatological Acute Clinics (VRA) has also commented on this issue.

Für den Leistungsbereich Rheumatologie erfolgte eine Ausgestaltung von Leistungsgruppen, die Relevanz für die akutstationäre rheumatologische Versorgung haben. Es wird auf die steigende Prävalenz entzündlich rheumatischer Erkrankungen hingewiesen. Darüber hinaus werden die Notwendigkeit von Strukturkriterien und die Vorhaltung eines Qualitätsmanagements für die hoch qualifizierte akutstationäre Versorgung von Menschen mit Rheuma in Deutschland beschrieben. Für die rheumatologische Versorgung in Deutschland werden flächendeckend hoch qualifizierte Fachärzte benötigt, die die ganze Bandbreite muskuloskeletaler Erkrankungen – vom „Leistungsbereich Bewegungsapparat“ bis hin zu komplexen Autoimmunerkrankungen mit drohender Organbeteiligung (Leistungsgruppen) – beherrschen und eine hervorragende Versorgung garantieren.

Das Ministerium für Arbeit, Gesundheit und Soziales (MAGS) in NRW hat im September 2019 ein vom MAGS in Auftrag gegebenes Gutachten zur Krankenhausplanung veröffentlicht [1]. Darin wurde festgestellt, dass die bisherige Krankenhausplanung zu einer Fehlentwicklung in der Krankenhauslandschaft geführt hat. Daher wird eine grundlegende Reform der Krankenhausplanung empfohlen. Statt einer als unzureichend beurteilten Beplanung von Bettenzahlen soll nun eine Bedarfsplanung folgen auf Grundlage einer detaillierten Ausweisung von Leistungsbereichen und Leistungsgruppen. Ziele sind u. a. die Erreichbarkeit eines Krankenhauses innerhalb vom 30 min, Ausschöpfung des Ambulantisierungspotenzials sowie Spezialisierung und Konzentration von Leistungen.

In einem nächsten Schritt hat das MAGS nun die Fachgesellschaften angeschrieben und anhand eines Fragenkatalogs um die leistungs-, bedarfs- und qualitätsorientierte Darstellung der stationären Leistung gebeten.

Von der geplanten Krankenhausreform ist auch die akutstationäre Rheumatologie betroffen. Entsprechend wurde die DGRh aufgefordert, für den Leistungsbereich Rheumatologie (akutstationär) eine Ausgestaltung der Leistungsgruppen zu erstellen, wie z. B. der multimodalen rheumatologischen Komplexbehandlung (OPS-8-983) [2, 3], welche die akutstationären Versorgungsinhalte der Rheumatologie sachgerecht abbildet. Vorgaben waren u. a. die Zuordnung von Qualitätskriterien zu einzelnen Leistungsgruppen sowie die stringente Berücksichtigung der Weiterbildungsordnungen der nordrhein-westfälischen Ärztekammern bei der Beantwortung eines Fragenkatalogs (s. Tabelle zur Zuordnung von Qualitätsvorgaben für Leistungsgruppen [Beispiele] im elektronischen Zusatzmaterial online).

Die zentralen Aussagen der von DGRh und VRA gemeinsam erstellten Stellungnahme sollen im Folgenden zusammengefasst werden – dies nicht zuletzt deshalb, weil ähnliche Reformbemühungen auch in anderen Bundesländern zu erwarten sind und daher die Zukunft der deutschen Rheumatologie insgesamt betreffen werden.

## Stationärer Behandlungsbedarf

Das MAGS-Gutachten stellt fest, dass 2,1 % der Bürger Nordrhein-Westfalens an entzündlich rheumatischen Erkrankungen leiden [4]. Diese Menschen sollen auch zukünftig gut versorgt werden: nicht nur zu ihrem eigenen Wohl, sondern auch zur Reduktion von großen volkswirtschaftlichen Belastungen. Bezüglich des akutrheumatologischen stationären Behandlungsbedarfs findet sich in Tab. 139 (Prognose-Kennzahlen nach Leistungsgruppen [LG] geordnet im Jahresvergleich [2017, 2032] – Leistungsbereich Bewegungsapparat und LG Rheumatologie) eine Fallzahl von 28,5/1000 Menschen für 2017 abnehmend nach 2032 auf 25,7/1000 Menschen, was begründet wird mit einer Verweildauerverkürzung von 8 auf 6,8 Tage im Mittel und einem Ambulantisierungspotenzial von 11,1 % in 2032. Diese Prognose lässt einige wesentliche Aspekte außer Acht, die wir nachstehend erläutern werden.

Die in dem Gutachten postulierte Abnahme der akutstationären Versorgungsquote berücksichtigt nicht die steigende Prävalenz entzündlich rheumatischer Erkrankungen:

1. Durch die moderne rheumatologische Therapie normalisiert sich die Lebenserwartung der Rheumapatienten um durchschnittlich 7 bis 10 Jahre.
2. Der Anteil älterer Menschen insgesamt (65 Jahre und älter) an der Bevölkerung steigt (https://www.lzg.nrw.de/ges_bericht/factsheets/bevoelkerung/index.html) und damit überproportional auch die Zahl der Menschen mit entzündlich rheumatischen Erkrankungen.
3. Ein Drittel aller Menschen mit Rheumaerkrankungen, die bisher in anderen Leistungsbereichen fachfremd versorgt werden [5], hat Anspruch auf eine *fachspezifische rheumatologische Versorgung*, damit sie nicht durch zu späte und zum Teil unrichtige Diagnose und Therapie geschädigt werden.

Die akutstationäre Versorgung von Menschen mit entzündlichen rheumatischen Erkrankungen soll entsprechend den 2011 veröffentlichten Strukturkriterien in einer eigenständigen Rheumaabteilung (Kriterium 1) erfolgen, die von mindestens 2 hauptamtlichen Rheumatologen (Kriterium 2) geleitet wird. Für eine solche Fachabteilung ist die jährliche Mindestmenge von >500 akutstationär versorgten Rheumafällen (davon mehr als 50 % mit entzündlich rheumatischen Erkrankungen) ein Qualitätsindikator (Strukturkriterium 5) [6]; sie benötigt ein multidisziplinäres Behandlungsteam (Kriterium 6) wie auch eine enge Kooperation mit anderen Fachabteilungen/Leistungsbereichen (Kriterium 7), auch standortübergreifend. Diese rheumatologischen Fachabteilungen/Fachkrankenhäuser sollten ein Qualitätsmanagement durchführen, wie z. B. KOBRA („Kontinuierliches Outcome Benchmarking in der akutstationären Rheumatologie“) [7], das vom Verband Rheumatologischer Akutkliniken (VRA) bereits seit 2012 konsequent angeboten wird.

## Zuordnung des Leistungsbereichs Rheumatologie

Im vorliegenden Gutachten zur Krankenhausplanung NRW wurde der Leistungsbereich Rheumatologie gemeinsam mit der Orthopädie und Unfallchirurgie dem „Leistungsbereich Bewegungsapparat“ zugeordnet. Dies führt aber zu Fehleinschätzungen, denn die Abgrenzung zu orthopädischen Krankheitsbildern ist v. a. ein Aufgabenschwerpunkt der Tätigkeit von internistischen Rheumatologen. In diesem Zusammenhang wird auf die in der ASV abgebildeten Rheumaerkrankungen verwiesen – hier wird das Kernteam von einem internistischen Rheumatologen geleitet.

Andere Leistungsbereiche sind Fächer wie Dermatologie, Nephrologie, Pneumologie und andere, die Mitglieder dieses Teams sind. Als ausgebildeter Internist ist der Rheumatologe mit der Fachexpertise ausgestattet, Menschen mit meist chronisch verlaufenden entzündlich rheumatischen Erkrankungen umfänglich zu versorgen. Hierbei sollte er/sie auch akutstationär die Federführung haben, wenn Rheumapatienten aufgrund einer zusätzlichen Organbeteiligung in organspezifischen Fächern wie Nephrologie, Pneumologie oder Kardiologie versorgt werden. Die dort erhobenen diagnostischen Befunddaten und die sich daraus ergebenden Therapieansätze müssen immer im Gesamtkontext der Versorgung von Rheumapatienten gesehen werden. Eine entsprechende rechtzeitige volle inhaltliche Einbindung des internistischen Rheumatologen unterstützt die frühzeitige Weichenstellung im Sinne optimaler Behandlungskonzepte, wodurch Krankheitsverlauf, Prognose, Lebensqualität und Arbeitsfähigkeit von Menschen mit rheumatischen Erkrankungen im Sinne besserer Outcomes beeinflusst werden können. Die Rheumatologie unter Einbeziehung der klinischen Immunologie ist ein internistisches Querschnittsfach mit Bezug zu zahlreichen anderen medizinischen Disziplinen, das in einem eigenen Leistungsbereich behandelt werden sollte.

Ein wichtiges positives Signal für die Arbeit von Rheumafachklinken und -abteilungen in Deutschland stellen die im März 2020 im Bundesanzeiger veröffentlichten Zentrumsregelungen des G‑BA für die Rheumatologie dar [8]. Hier wird den akutstationären rheumatologischen Versorgungseinrichtungen bei Erfüllung entsprechender Mindestkriterien als Rheumazentrum eine Leuchtturmfunktion gegeben.

## Ambulantisierungspotenzial

Die stationäre Rheumatologie versorgt multimorbide, in ihrer Körperfunktion deutlich eingeschränkte Menschen mit zum Teil seltenen, häufig komplexen Erkrankungen, deren Verlauf durch Infektionen – infolge der notwendigen intensiven Therapie – kompliziert wird. Es werden mehr als 80 % der in rheumatologischer Weiterbildung befindlichen Fachärzte in rheumatologischen Akutkliniken, Fachabteilungen und Unikliniken ausgebildet. Die Weiterbildung muss unter Berücksichtigung der Weiterbildungsordnung größtenteils stationär erfolgen, u. a. um die akutstationäre Versorgung von Menschen mit komplexen entzündlich rheumatischen Erkrankungen zu erlernen. Diese Leistungen können keineswegs, wie im Gutachten behauptet „in der Grund- und ambulanten Versorgung“ (S. 330 oben) abgebildet werden.

Mit den § 116b-Ambulanzen wie auch den neuen ASV-Ambulanzen nach SGB V [9] hat die deutsche Rheumatologie – vertreten durch DGRh, VRA und BDRh – wesentliche Meilensteine im Sinne der Ambulantisierung bewegt und nicht nur die sektorale Patientenversorgung gefördert, sondern bereits in der letzten Dekade die ambulante Patientenversorgung erheblich intensiviert [10]. Dies ist angesichts der nur wenig wachsenden Zahl internistisch rheumatologischer Fachärzte auch dringend erforderlich.

Eine Lösung kann hier nur sein, weitere akutstationäre Facharztausbildungsstellen einzurichten, um das vom G‑BA gesetzte Ziel zu erreichen, einen Anteil von 8‑ bis 10 % am Spektrum der internistischen Fachrichtungen mit Fachärzten für Rheumatologie als Vertragsärzte zu besetzen.

Für die Wahrnehmung der akutstationären Rheumatologie in Deutschland wäre es hilfreich, wenn auch in NRW weitere medizinische Fakultäten über eine Hauptabteilung für Rheumatologie verfügen würden. Hier hat sich mit den universitär geschaffenen Strukturen in Bochum und Bielefeld zuletzt schon eine positive Entwicklung gezeigt.

Die rheumatologische „Kurort-Medizin“ ist schon lange Vergangenheit und nur noch in Museen zu finden. Die rheumatologische Versorgung erfolgt durch hoch qualifizierte Fachärzte, die die ganze Bandbreite muskuloskeletaler Erkrankungen vom Leistungsbereich Bewegungsapparat bis hin zu komplexen Autoimmunerkrankungen (verschiedene Leistungsgruppen) mit drohender Organbeteiligung beherrschen und eine hervorragende Versorgung garantieren:

Die Zielsetzung der Krankenhausplanung NRW 2020 ist im Schwerpunkt die Spezialisierung und Konzentration von Leistungen, um durch diesen Schritt die akutstationäre Versorgungsqualität für alle Fachrichtungen zu stärken. Der Leistungsbereich Rheumatologie in Deutschland hat in den letzten beiden Jahrzehnten Qualitätssicherung und Qualitätsmanagement in allen Versorgungssektoren nachhaltig gefördert, beispielhaft sind einige Publikationen nachstehend aufgeführt:

- Braun J, Schneider M, Lakomek H.-J. Eckpfeiler der Qualitätssicherung in der Medizin in Deutschland – Wichtige Impulse für die rheumatologische Versorgungssituation. Z Rheumatol 2016; 75:203–212
- Braun J, Bessler F, Lakomek H.-J., Rudwaleit M. Internationale Qualitätsindikatoren in der Rheumatologie – Vorschläge für die Rheumatoide Arthritis. Z Rheumatol 2016; 75:330–337
- Braun J, Krause A, Aringer M, Burmester G, Bessler F, Engel J.-M., Faubel U, Fischer-Betz R, Gromnica-Ihle E, Hellmich B, Kötter I, Krüger K, Lakomek H.-J, Lorenz H.-M., Manger B, Märker-Hermann E, Minden K, Müller-Ladner U, Rautenstrauch J, Rehart S, Riemekasten G, Rudwaleit M, Rüther W, Schett G, Schuch F, Schulze-Koops H, Specker C, Wassenberg S, Wiek D, Zink A, Schneider M. Europäische Versorgungsstandards für Menschen mit rheumatoider Arthritis. Übersetzung und Kommentierung der von der EULAR unterstützten Vorschläge des eumusc.net durch eine Task Force von DGRh und VRA mit Unterstützung der Deutschen Rheuma-Liga. Z Rheumatol 2016;75:416–428
- „Treat-to-Target“ (T2T) aus der Sicht der stationären Rheumatologie. Lakomek H.-J, Krause A. Z Rheumatol 2011; 70:656–663

Diese fachbezogenen Qualitätsbausteine können darüber hinaus die Zielsetzung der geforderten Qualitätsmessungen innerhalb der Mindestkriterien von zukünftigen Rheumazentren [8] nachhaltig unterstützen.

## Supplementary Information


