# Supplementary material for: Future of acute inpatient rheumatology in Germany: Statement of the Boards of the German Society for Rheumatology and the Association of Rheumatological Acute Clinics on hospital planning North-Rhine/Westphalia 2019 for the discipline rheumatology
Source: Z Rheumatol. 2020 Dec 12;80(1):103–6. [Article in German] doi: 10.1007/s00393-020-00939-4 (PMC7872996; doi:10.1007/s00393-020-00939-4)
Supplement: Supplementary file 1 [file 393_2020_939_MOESM1_ESM.pdf]

Zusatzmaterial

Tabelle zur Zuordnung von Qualitätskriterien

Leistungsbereich Rheumatologie										
**Leistungsgruppe**		**Erbringung verwandter LG**		**Vorhaltung Geräte**	**Fachärztliche Vorgaben**		**Sonstige Struktur- und Prozesskriterien**	**Mindestmengenvorgaben**		**OPS und/oder ICD**
		Standort	Kooperation		Qualifikation	Verfügbarkeit		ja/nein	wenn ja, welche?	
Großgefäßvaskulitiden	Mindest-voraussetzung	Rheumatologie		Farb-Duplexsonographie	FA Rheumatologie	2 FÄ, davon eine/r 24/7	Fast-Track-Diagnostik ink. Farbduplexsonographie innerhlab von 24 Stunden, Verfügbarkeit Biopsie der Temporalarterien, Zugang zu bildgebender Großgeräte-Diagnostik der extrakraniellen Gefäße (MRT und/oder PET-CT), akutstationäre Strukturqualität (Lakomek et al, 2011)	nein	s. Appendix	M31.5, M31.6, M31.4, OPS-8-983
	Auswahl-kriterium		Radiologie, Nuklearmedizin, Ophthalmologie, Pathologie	MRT-Angiographie, PET-CT, CT-Angiographie						
Vaskulitiden kleiner und mittelgroßer Gefäße	Mindest-voraussetzung	Rheumatologie		Farbduplex-Sonographie, Sonographie	FA Rheumatologie	2 FÄ, davon eine/r 24/7	Mikroskopische Urinuntersuchung am Standort, Ergebnis Autoimmunserologie (PR3-ANCA, MPO-ANCA, GBM-Antikörper) innerhalb von 24 Stunden, akutstationäre Strukturqualität (Lakomek et al, 2011)	nein	s. Appendix	M30.0, M30.1, M30.2, M30.8, M30.1, M35.2, M31.3, M31.7 L95.8, D89.1, D69, OPS 8-983
	Auswahl-kriterium		Nephrologie, Pneumologie, Neurologie, Ophthalmologie, Radiologie, Intensivmedizin	Endoskopie, CT, MRT			Plasmapherese (für Kryoglobulinämie, anti-GBM-Erkrankung) und Intensivmedizin in Kooperation			
Rheumatodie Arthritis	Mindest-voraussetzung	Rheumatologie		Farb-Duplexsonographie, Sonographie	FA Rheumatologie	2 FÄ, davon eine/r 24/7	Physiotherapie, Ergotherapie, Psychologie, multimodale rheumatologische Komplextherapie (OPS 8-983), akutstationäre Strukturqualität (Lakomek et al, 2011)	nein	s. Appendix	M05.00; M06.00, OPS 8-983, OPS 8-020.4, OPS 8-020.5, OPS 8-158, OPS 8-159, OPS 6-001/2/3/5/6/9/a, OPS 8-983; OPS-1-854; OPS-8-020.4,5
	Auswahl-kriterium		Radiologie, Pneumologie, Orthopädie	CT, MRT, Bronchoskopie, DXA-Messplatz						
Kollagenosen	Mindest-voraussetzung	Rheumatologie		Sonographie, Farbduplexsonographie, Kapillarmikroskopie	FA Rheumatologie	2 FÄ, davon eine/r 24/7	Sonographien innerhalb von 24 Stunden, Verfügbarkeit Biopsie der Muskulatur, Verfügbarkeit Biopsie Haut, Biopsie N. Suralis, Biopsie Niere, Zugang zu bildgebender Großgeräte-Diagnostik (CT, MRT, ggf auch PET), enge Zusammenarbeit mit den kooperierenden Fachgebiteten für Diagnostik und Therapie, Physio- und Physikalische Therapie, , Ergotherapie	nein	s. Appendix	M 32.0, M32.1. M32.8; M34.0, M34.1, M34.2, M34.8; M35.4; M33.0, M33.1, M33.2; M36.0, M35.1, M35.0; zahlreiche *Diagnosen und Nebendiagnosen, OPS: 8-54, 6-00, 8-561, 8-983; u.v.a.m.
	Auswahl-kriterium		Immunlabor, Nephrologie, Pulmonologie, Neurologie, Radiologie, Gastroenterologie, Angiologie oder Gefässchirurgie, (HNO, Ophthalmologie), Intensivmedizin, Schmerztherapie	Echo, MRT, CT (PET), EKG, Bodyplethysmographie, LZ-RR und -EKG,						
Spondyloarthritiden	Mindest-voraussetzung	Rheumatologie		Farb-Duplexsonographie, Sonographie	FA Rheumatologie	2 FÄ, davon eine/r 24/7	Physiotherapie, Ergotherapie, Psychologie, multimodale rheumatologische Komplextherapie (OPS 8-983), akutstationäre Strukturqualität (Lakomek et al, 2011)	nein	s. Appendix	OPS 8-983, M46.8, M45.0, 8-020.4, 5, OPS-1-854
	Auswahl-kriterium		Radiologie, Unfallchirurgie, Orthopädie	CT, MRT						
multimodale rheumatologische Komplextherapie	Mindest-voraussetzung	Rheumatologie			FA Rheumatologie	2 FÄ, davon eine/r 24/7	Physiotherapie, Ergotherapie, Psychologie, Schmerztherapie	nein	s. Appendix	OPS 8-983, OPS 8-986
	Auswahl-kriterium									

Appendix:

Die Festlegung von Mindestmengen einzelner Leistungsgruppen wird von den Rheuma-Experten nicht befürwortet. In 2011 hat der Verband Rheumatologischer Akutkliniken (VRA) gemeinsam mit Vorstandsmitgliedern der Deutschen Gesellschaft für Rheumatologie (DGRh) die "Strukturqualität" für akutstationäre Rheuma-Einrichtungen festgelegt (s. Tabelle). Hier wurde die Gesamtzahl akutstationär zu versorgender Menschen mit Rheuma auf mehr als 500 Fälle jährlich festgelegt, wobei der Anteil der entzündlich-rheumatischen Erkrankungen der Gelenke, der Wirbelsäule und des Bindegewebes mehr als 50 % betragen soll (Kriterium 5) [Lakomek et. al., 2011]
